# Hidden dynamics of economic hardship: Characterizing economic unpredictability and its role on self-regulation in early childhood

**DOI:** 10.1017/S0954579425100771

**Published:** 2025-10-29

**Authors:** Meriah L. DeJoseph, Nicole Walasek, Sihong Liu, Ethan S. Young, Abbie Raikes, Marcus Waldman, Willem E. Frankenhuis, Philip Fisher

**Affiliations:** 1 Stanford Center on Early Childhood, Graduate School of Education, Stanford University, Stanford, CA, USA; 2 Evolutionary and Population Biology, Institute for Biodiversity and Ecosystem Dynamics, University of Amsterdam, Amsterdam, Netherlands; 3 Department of Psychology, Utrecht University, Utrecht, Netherlands; 4 College of Public Health, University of Nebraska Medical Center, Omaha, NE, USA; 5 Max Planck Institute for the Study of Crime, Security, and Law, Freiburg, Germany

**Keywords:** Economic hardship, environmental unpredictability, self-regulation, early childhood

## Abstract

Economic hardship is known to shape children’s self-regulation, yet little is understood about how fluctuations in hardship unfold over time and whether different patterns of unpredictability carry unique developmental consequences. Using a socioeconomically diverse sample, we tracked families’ subjective economic hardship across 15–36 monthly assessments and applied an environmental statistics framework to quantify four indices of unpredictability: changepoints in mean, changepoints in variance, coefficient of variation, and noise. PCA identified two distinct forms of economic unpredictability: one marked by frequent, unpredictable hardship, and another by infrequent but abrupt hardship. Economic unpredictability was disproportionately experienced by racially minoritized and lower-income families in our sample, reinforcing structural inequities in economic resources. Relations between these indices and caregiver-reported measures of family routines and day-to-day unpredictability were weak, suggesting wide heterogeneity in the ways families adapt to economic unpredictability. Leveraging propensity score methods, we isolated the effects of unpredictability from hardship severity, finding that both were associated with greater self-regulation challenges in early childhood, with the strongest effects for hardship severity. These findings underscore the importance of capturing economic hardship as a dynamic and multidimensional experience, with implications for policy efforts aimed at promoting stability in families’ access to resources over time.

Children’s earliest experiences guide the development of basic cognitive, emotional, and behavioral regulation skills that set the stage for long-term outcomes. A growing literature on the role of poverty has revealed complex socioeconomic influences on how children adaptively self-regulate (i.e., modulate their behavioral, cognitive, and emotional responses) across various contexts (Blair & Raver, 2012; DeJoseph & Ellwood-Lowe et al., 2024). Children growing up in poverty are more likely to experience intersecting constraints and challenges such as a lack of material resources, unstable housing, and higher levels of exposure to violence (Duncan, 2021; Duncan & Magnuson, 2013). Although many children flourish in the face of stress that economic hardship brings, at a population level, chronic and unpredictable poverty-related adversities put children at an increased risk of developing self-regulation challenges. These challenges, while potentially adaptive in the immediate context of poverty (e.g., steeper future discounting in unpredictable conditions), may disrupt their capacity to meet the learning and psychosocial demands of formal schooling and beyond (Blair & Raver, 2015). Clarifying the environmental determinants that explain associations between poverty and child self-regulation are central for the creation of programmatic efforts that address families’ challenges while leveraging extant strengths.

Moreover, despite the established link between poverty and self-regulation, how poverty exerts its influence remains largely unknown. This is in part because poverty is often treated as a unidimensional construct, which may obscure the underlying mechanisms by which it operates. The type and temporal variability of poverty-related experiences, and their role on self-regulation development in early childhood, may be candidate mechanisms underlying the effects of child poverty on long-term outcomes. Here we focus on one such type of experience, subjective perceptions of economic hardship and their corresponding temporal variability over time. Specifically, we draw upon a recently developed framework (Walasek et al., 2024) to descriptively characterize economic unpredictability in early childhood and examine its role in children’s emerging self-regulation skills.

## Economic hardship and self-regulation in early childhood

The first five years of a child’s life are pivotal for the emergence of self-regulatory skills (e.g., inhibitory control, attention, emotional responsivity) that lay the foundation for lifelong thriving. Substantial neurodevelopmental changes occurring during this period are associated with increased behavioral and emotional regulation in the context of environmental demands (Blair & Ku, 2022). These early regulatory skills are critical for school readiness (Blair & Raver, 2015), and predict a host of mental and physical health outcomes (Howard et al., 2018; Robson et al., 2020). Importantly, self-regulation development is deeply embedded in – and therefore shaped by – environmental context (Miller-Cotto et al., 2022). Thus, individual differences in self-regulation are in large part due to children’s early experiences. Enhanced neuroplasticity in early childhood makes emerging cognitive, behavioral, and emotional regulatory systems particularly sensitive to such life experiences (Greenough et al., 1987; Takesian & Hensch, 2013).

Effects of child poverty on developing regulatory systems are present as early as infancy, with more years spent at or below the federal poverty line predicting greater self-regulation challenges over time (Burchinal et al., 2018; Raver et al., 2013). Additional work has shown that the additive culmination of poverty-related adversities (i.e., cumulative risk) alters the regulation of the stress response system, which can in turn change how children self-regulate (Blair et al., 2011; Palacios-Barrios & Hanson, 2019). The widespread use of income and cumulative risk scores have provided foundational insights into the consequences of child poverty (Jenson et al., 2017) that have been relevant in both scientific and policy contexts. However, both approaches also have limitations. In particular, income relies on somewhat coarse and distal proxies of lived experience; in contrast, cumulative risk assumes that all adverse experiences additively combine in equally weighted and linear ways. Although these approaches have established that poverty is broadly associated with self-regulation, open questions remain about *when* and *how* experiences in the context of poverty unfold to shape early individual differences in self-regulation.

Here we draw upon several contemporary frameworks of early-life adversity (Frankenhuis et al., 2020; Ellis et al., 2022; DeJoseph & Ellwood-Lowe et al., 2024; Walasek et al., 2024) that afford a well-rounded approach for revealing nuanced links between poverty and self-regulation. Collectively, these frameworks call for direct measures of child poverty that move beyond income or cumulative risk and toward characterizing the *type* and *temporal features* of proximal, poverty-related experiences. Such characterizations align with recent paradigms used to study the impact of early-life unpredictability, teased apart from mean levels of adversity, on the neurobiological systems of humans (Farkas & Jacquet, 2024; Glynn et al., 2019; Lancaster & Wass, 2024; Munakata et al., 2023) and non-human animals (Baram et al., 2012). In the current study, we focus on the role of economic hardship, as indexed by caregivers’ reports of a lack of access to basic necessities (e.g., food, housing, healthcare).

Exposure to economic hardship – and accompanying lack of educational and material resources – may constrain opportunities for learning that impact neurodevelopment. Compared to children from affluent families, children in homes grappling with resource scarcity may have less access to enriching sensory, cognitive, and social stimulation (Rosen et al., 2020; Votruba-Drzal, 2003). Such reduced sociocognitive stimulation may alter the structure and function of brain regions underlying cognition, exerting downstream effects on self-regulatory behaviors that depend on these brain areas (Rakesh et al., 2023; Rosen et al., 2018). It is important to note that while economic constraints may limit certain types of stimulation, other forms may be present (e.g., rather than language exposure via books children may be exposed to enriching language around a cultural tradition or family mealtimes), shaping development in ways that are adaptive to these specific environments (DeJoseph & Ellwood-Lowe et al., 2024).

Although exposure to economic hardship can be severe and enduring for some children, many also experience volatile fluctuations in economic circumstances over time (Magnuson & Votruba-Drzal, 2009; Hardy & Zilliak, 2014). Indeed, more than one third of households in the U.S. experience significant shifts in household income within a single year (Dynan et al., 2012) – economic instability further exacerbated by the recent COVID-19 pandemic (Liu et al., 2022a). One of the hallmarks of the pandemic was how it led to widespread economic instability as caregivers lost their jobs, childcare facilities shut down, food insecurity rose, and family routines and other hardships were drastically altered (Menasce Horowitz et al., 2020).

Recent extensions of contemporary models of adversity assert that in addition to mean levels in adverse experiences, the level of random variation over time, or *unpredictability*, matters for understanding how such experience shapes children’s development (Ellis et al., 2022, 2009; Liu et al., 2022). Unpredictability poses a different set of learning and regulatory demands on a child than when adversity is consistent (Ugarte & Hastings, 2024), and may therefore lead to different neurodevelopmental and behavioral responses. More specifically, children may detect and respond to (1) specific cues indicating high environmental unpredictability across evolutionary time (e.g., residential changes), and/or (2) the statistical structure of the environment, such as stability and change in economic hardship (for a review, see Young et al., 2020). Through these cues, patterns of lived experiences are summarized and revised through neural mechanisms that use these estimates to guide learning and behavior in adaptive ways (Young et al., 2020). For example, prior research has shown intriguing patterns of cognitive skills that are both enhanced and impaired in the context of unpredictable environments. This research suggests that unpredictability may enhance some aspects of attention shifting and working memory, albeit at the cost of impaired inhibitory control (Fields et al., 2021; Mittal et al., 2015; Young et al., 2018; see also Howard et al., 2020; Nweze et al., 2021; although cognitive modeling studies have found neither of these effects, Vermeent et al., 2024, 2025); though these studies used different measures of unpredictability, limiting inferences across them. Nevertheless, the ability to flexibly detect threats and fleeting opportunities (via enhanced attention shifting) and track changing environmental conditions (via enhanced working memory) might confer greater adaptive benefits than inhibiting dominant or instinctual responses (however, see Lucon-Xixxato et al., 2023; Tello-Ramos et al., 2019). Importantly, this emerging line of research comes exclusively from adolescent and adult samples, and highlights the need to examine these links earlier in development, when self-regulatory systems are most malleable in response to experience.

## Methodological advancements in operationalizing the dynamic unfolding of economic hardship: Formalizing and isolating the role of unpredictability

Whether and how self-regulation in early childhood varies based on the predictability of dimensions of adversity remains a pressing empirical question. Despite the rapidly growing literature on environmental unpredictability (and the recent unpredictability of life itself), conceptual and methodological challenges persist. These challenges make it difficult to capture and empirically isolate the role of economic unpredictability. One key challenge is the myriad ways that unpredictability can be operationalized. A recent review noted that of the 21 studies published on environmental unpredictability since 2020, 15 different measures were used across multiple report formats (Young et al., 2020).

In the child poverty literature, unpredictability has been indexed as the number of residential moves or caregiver changes (e.g., Roy et al., 2014; Young et al., 2024), caregiver- or assessor-reported household chaos (e.g., Evans et al., 2005; Garrett-Peters et al., 2016; Raver et al., 2015), the number of transitions in and out of poverty (Raver et al., 2013), residual variance (e.g., variance around individual regression lines) of income (Li et al., 2018; Li & Belsky, 2022), standard deviation of income and neighborhood disadvantage (Young et al., 2024), coefficient of variance (i.e., standard deviation divided by the mean) of subjective material hardship (Liu et al., 2023), and intra-class correlations of objective and subjective reports of economic hardship (Miller et al., 2025). Other studies have focused on broader characterizations of environmental unpredictability in day-to-day activities (e.g., Davis et al., 2024; Glynn et al., 2019), caregiving (e.g., Ugarte & Hastings, 2024), and noise exposure (Wass et al., 2019). Collectively, these studies have shown that greater unpredictability in income, household chaos, or housing and caregiving instability tends to increase risk for negative outcomes across a range of social and cognitive domains. However, the wide variation in the measurement of unpredictability hinders the ability to make direct comparisons across studies, which leads to conceptual ambiguity and limits theoretical validation (Flake & Fried, 2020; Frankenhuis et al., 2023). Even more importantly, the small number of longitudinal time points represented, most of which are less than six, may be obscuring the actual levels of economic unpredictability children experience. Densely sampled longitudinal data – which the current study leverages – are needed to accurately quantify the statistical properties of a dynamically unfolding environmental experience (Ehlman et al., 2023; Kievit et al., 2022; Lancaster & Wass, 2024).

A recently developed environmental statistics framework provides clear and quantifiable statistical operationalizations of unpredictability that can be applied to densely sampled data (Walasek et al., 2024). The framework decomposes environmental variation in individual time-series data into *distributional* (e.g., mean, variance/standard deviation) and *dynamic* statistics (e.g., autocorrelation, changepoints). Compared to distributional statistics, in which the order of the time series does not matter, dynamic statistics describe order-dependent patterns of stability and change over time. Both distributional and dynamic statistics reflect discrete properties of the environment that might be (implicitly) detected and responded to via statistical learning mechanisms that guide self-regulation development (Schapiro & Turk-Browne, 2015).

Here we consider a rich set of indicators thought to most comprehensively capture the experience of economic unpredictability, which we define as random variation in economic hardship over time. These include indices capturing the *amount* (i.e. frequency and magnitude) of instability (e.g., coefficient of variation, changepoints in mean and variance) and *patterns* of irregularity (e.g., noise, or what is left in the time series after accounting for systematic changes) in economic conditions. It is important to note that a range of other approaches exist for quantifying unpredictability – such as methods applied to discrete signals (Davis & Glynn, 2024; Ugarte & Hastings, 2024), interactions between children and caregivers (Lancaster & Wass, 2024), or statistical threat models (Farkas et al., 2024). We choose to follow the approach laid out in the environmental statistics framework (Walasek et al., 2024) as it offers a formalized yet flexible way of capturing a broad range of experiences important for development. Because the current study is one of the first to adopt this framework, and the first to adopt it in the investigation of economic hardship, we take an exploratory (rather than confirmatory) approach to examining the utility of these unpredictability indices. In other words, we do not a priori favor any one hypothesis; rather our goal is to build greater conceptual and theoretical clarity in this nascent area of research. Specifically, in addition to examining links with self-regulation outcomes, we explore whether these indices are correlated with one another, indicate the same underlying construct, and whether patterns qualitatively differ across key sociodemographic characteristics.

## The present study

Using data from a large national U.S. survey of socioeconomically diverse families with children ages 0–5 years old collected during and after the COVID-19 pandemic, we leveraged contemporary theoretical frameworks and quasi-experimental methods to address two primary aims. The first aim was to characterize economic unpredictability in early childhood. Specifically, we drew upon a novel environmental statistics framework (Walasek et al., 2024) to generate indices capturing dynamic fluctuations in over 15 repeated measures of caregiver-reported economic hardship collected between 2020–2023 – a salient time of economic instability and resource uncertainty, in part due to the COVID-19 pandemic. Given the lack of prior research in this domain, we descriptively explored relations between the unpredictability indices, differences across racial/ethnic groups and SES, as well as relations with previously used subjective measures of unpredictability. Data reduction techniques that optimize shared variance (i.e., PCA) were used to examine whether economic unpredictability was best captured as a singular or multiple component dimension.

Although our first aim was exploratory, we anticipated that families from lower-income and racially marginalized backgrounds would show greater mean levels of economic hardship as well as greater unpredictability. This pattern would align with prior research showing longstanding economic disparities between these groups, which has been exacerbated in recent years amidst the pandemic (Liu & Fisher, 2022; Parolin, 2021). We also anticipated negative relations between economic unpredictability and other subjective measures of family routines, as volatile disruptions in financial circumstances likely exert downstream effects into families’ day-to-day activities (Evans & Wachs, 2010).

Our second aim was to determine the effect of both economic hardship (in terms of average levels indexing severity) and economic unpredictability on self-regulation outcomes in early childhood. To do this, we used propensity score weighting to isolate the links between dimensions of economic hardship and unpredictability on caregiver-reported child self-regulation. This quasi-experimental method balances confounding factors across levels of each environmental exposure, simulating the conditions of random assignment. Put simply, it allows us to make comparisons as if we had randomly assigned children to different levels of economic conditions. In turn, this approach afforded the opportunity for critical insights into whether overall levels of economic hardship, or the dimensions of economic unpredictability derived from our first aim, are differentially related to early self-regulation skills. While we expected that greater levels of both economic hardship and unpredictability will be broadly associated with greater self-regulation challenges, we did not have a priori expectations about the relative magnitudes of these relations.

## Methods

### Participants

Participants were drawn from the Rapid Assessment of Pandemic Impact on Development (RAPID; see Fisher et al., 2024; Liu et al., 2023). The RAPID project is an ongoing national survey of caregivers with children ages 0–5 years that includes measurements of family experiences throughout the pandemic and beyond. In the current study, we used data collected from April 2020 to August 2023. Our analysis was limited to participants who completed a minimum of 15 monthly responses of economic hardship – a recommended lower bound to generate reliable unpredictability statistics (see Walasek et al., 2024) outlined in our first aim. This resulted in an analytic sample of 321 participants for aim 1, whose responses to the economic hardship measure ranged from 15 to 36.

For aim 2, the sample from aim 1 was further limited to participants who also completed a one-time follow-up survey collected in August 2023 reporting on their child’s self-regulation (mean child age = 3.32 years old; sd = 1.40). A total of 125 families made up this subsample.

A demographic breakdown of our analytic sample for aim 1 (*n* = 321) and aim 2 (*n* = 125) can be found in Table 1. Demographics of these two subsamples were comparable.

**Table 1.** Demographic characteristics of the study samples for aims 1 and 2

Demographic characteristics		Aim 1 sample (*n* = 321)		Aim 2 subsample (*n* = 125)	
		*n*	%	*n*	%
Caregiver race/ethnicity	Black	52	16.20%	21	16.20%
	Latine	66	20.56%	40	32%
	White	171	53.27%	55	44%
	Other	32	9.97%	9	7.20%
Caregiver highest education	High school diploma or GED	14	4.36%	5	4%
	Associate degree	30	9.35%	12	9.6%
	Some college	47	14.64%	23	18.40%
	Bachelor’s degree	109	33.96%	39	31.20%
	Master’s degree	76	23.68%	31	24.80%
	Doctorate/Professional degree	37	11.53%	12	9.60%
	Other/NA	8	2.49%	3	2.40%
Federal poverty level	Below 200%	104	32.40%	46	36.80%
	200 - 400%	100	31.15%	36	28.8%
	Above 400%	116	36.14%	43	34.4%
	Other/NA	1	0.31%	0	0%

### Procedure

The RAPID sampling method involved initial recruitment (baseline) and regular survey assessments (follow-ups), both conducted on an ongoing basis, initially following a weekly and later on a biweekly alternating schedule. Specifically, caregivers completed weekly surveys from April 2020 to July 2020, alternating biweekly from August 2020 to December 2021, and monthly afterwards. Each baseline recruitment involved caregivers expressing interest by filling out an eligibility survey. The criteria for ongoing survey participation required respondents to be: (1) at least 18 years old, (2) the primary caregiver for a child aged 0–5, (3) proficient in English and/or Spanish, and (4) residing in the United States. Eligible caregivers agreed to be recontacted for subsequent follow-ups, responded to essential survey questions (like demographics, economic hardship, well-being), and joined the participant pool. For the initial assessment, RAPID employed convenience sampling for broad recruitment, leading to a participant pool not aimed to be nationally representative.

Caregivers received email invitations to participate in follow-up surveys, which comprised both standard and unique questions (such as those about family conflict and routines, varying with each survey). For each of these follow-ups, 2000 caregivers were chosen through a random selection from the participant pool. The expected response rate was about 50%, aiming for roughly 1000 responses for each survey. Families were incentivized with $5 for completing each survey. Significant efforts were put into including a more diverse range of participants, especially from racially/ethnically diverse backgrounds and lower-income households. In each follow-up survey, the random selection from overall participant pool was stratified based on the participants’ race/ethnicity and poverty level (specifically, their percentage of the U.S. federal poverty level) to achieve a representation that reflects these demographic aspects nationally. This stratification also aimed for representativeness of geographical distribution across the U.S. According to this approach, the frequency of survey invitations and the number and timing of responses varied among families.

To minimize online survey fraud, surveys were manually and systematically inspected at baseline and all follow-up surveys based on I.P. address, attention check questions (e.g., What year is 10 years into the future?), and inconsistent data patterns to detect and remove fraudulent responses. Responses were identified as invalid if: (1) duplicated I.P., email address, or other identifiable information were found in previous baseline surveys, (2) I.P. address was identified as “survey farms” using an external online tool, (3) failure to answer the attention check questions correctly (within 1 digit error margin), and (4) inconsistent data pattern (e.g., reported child age in baseline survey did not match responses in the eligibility survey, reported caregiver gender did not match reported relationship to the child). These strategies were formed based on a series of recently developed fraud detection protocols that had proven effective (e.g., Ballard et al., 2019; Pozzar et al., 2020; Storozuk et al., 2020).

All study procedures were approved by Stanford University Institutional Review Board (#64791). See Fisher et al. (2024) for more information on the RAPID study.

### Measures

#### Economic hardship and unpredictability

Economic hardship and unpredictability were calculated via a 6-item economic hardship measure indexing the extent to which families reported being unable to pay for basic needs in the past month. This measure was adapted from the Institute of Medicine financial strain scale (IOM, 2014). Caregivers reported whether they had difficulty meeting needs. Specifically, they were asked, “Which of these needs have been hard to pay for in the past month?” and then responded with “1-Yes” or “0-No” to needs, including food, housing, utilities, healthcare, childcare, and socioemotional well-being.

For each survey response, scores were summed (ranging from 0–6), with higher scores indicating greater economic hardship. For participants who provided more than one response in a given month, a mean of available responses for that month was calculated. These monthly scores were then transformed by adding a one to each sum score (changing the range to 1–7). This transformation was required as certain statistics – specifically the coefficient of variation (see details below) – cannot be accurately calculated for participants who had a score of 0 across all or most time points. To index children’s overall exposure to economic hardship (i.e., hardship severity) across time, participants’ monthly scores were averaged across all available months to generate a cumulative count score (mean = 1.63, sd = 1.04).

Economic unpredictability was indexed via several indices as outlined in Walasek et al (2024) and outlined in Table 2. Specifically, each participant’s time series (min # months: 15; max # months: 36) of monthly economic hardship were used to generate four summary scores capturing different operationalizations of unpredictability: (1) *Coefficient of variation* (standard deviation divided by the mean) indexed the average fluctuations around mean levels of economic hardship, whereby greater values indicate more unpredictability. *Changepoints* indexed significant shifts in the (2) mean or (3) variance across the measurement period, whereby more changepoints indicate greater unpredictability. These three indices are thought to capture the *amount of instability* in economic conditions. (4) *Noise* indexed the extent to which noise in economic hardship (i.e., what is left in the time series after subtracting systematic patterns, such as seasonal patterns, as well as linear trends in the mean and variance) is correlated across time lags. Values closer to 0 indicate greater randomness, values closer to 2 indicate slower and more predictable change, and values between 0 and −1 indicate rapid but predictable change. Noise was additionally transformed so that higher values indicated higher unpredictability (see Table 2). In contrast to the former three indices (which capture *amount*), noise captures *patterns* of irregularity in economic conditions. Unless otherwise noted, we use and refer to the transformed noise variable throughout the remainder of the paper. Additional information on how each of these summary indices were calculated and used in subsequent analyses is discussed in the Analytic Strategy (section 2.4.1) below.

**Table 2.** Glossary of environmental statistics examined in the current study

Environmental statistic	Range	Meaning
Coefficient of variation	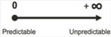	Extent of variability in economic hardship relative to overall mean levels
Changepoints in mean		Number of times when average economic hardship levels significantly shift, whereby more frequent shifts indicate greater economic unpredictability
Changepoints in variance		Number of substantial or abrupt changes in the variability of economic hardship, indicative of periods of heightened economic unpredictability
Noise	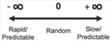 Raw value	Extent to which noise in economic hardship (i.e., participant-level time series after the removal of systematic patterns) shows predictable patterns
	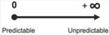 Transformed value	The reciprocal transformation of the absolute value of noise, such that higher levels indicate higher unpredictability

#### Child self-regulation

Children’s self-regulation was assessed via the 44-item caregiver-reported Global Scales of Early Development Psychosocial Form (Waldman et al., 2024). Items were chosen by a World Health Organization (WHO) committee of experts in developmental psychology and pediatrics to inform progress in promoting children’s psychosocial well-being at the population level. Items assessed aspects of emotional self-regulation (e.g., “*If your child gets upset, do they cry for a long time?”*), cognitive self-regulation (e.g., *“Does your child have trouble paying attention?”*), and behavioral self-regulation (e.g., *“Does your child act impulsively without thinking (e.g., running into the street without looking or doing other dangerous behaviors?”*). Caregivers reported the frequency with which their child exhibits behaviors on a 3-point Likert scale (0-“Never or Almost Never; 1-“Sometimes”, or 2-”Often”). The Global Scales of Early Development Psychosocial Form has recently demonstrated positive evidence of validity and reliability using a sample of 836 American children aged 180 days to 71 months (Waldman et al., 2024). Factor scores derived from the bi-factor measurement model presented in Waldman et al. (2024) were used to index children’s self-regulatory capacities (mean = 0.14, sd = 0.90). Higher scores indicate greater self-regulation challenges.

#### Questionnaire-based measures of environmental (un)predictability

Family routines and overall unpredictability in several aspects of children’s daily lives were assessed via two special module measures. Family routines were assessed several times between 2020–2023 and were composed of five items developed from the RAPID team to indicate the frequency of several day-to-day routines in the past month (e.g., *“Whole family ate dinner together almost every night.”*). Responses ranged from 1 - “Almost never” to 4 - “Almost every day.” Items exhibited acceptable internal consistency (*α* = .78) and were averaged across time to index overall family routines, with higher scores indicating greater predictability in family routines (mean = 3.26, sd = 0.48). Overall unpredictability was assessed once in June 2022, consisting of a measure composed of 18 items selected from the Questionnaire of Unpredictability in Childhood (QUIC; Glynn et al., 2019) that incorporated four subscales (i.e., physical environment, safety and security, parental environment, and parental predictability). Caregivers reported on typical experiences or the presence of characteristics relating to unpredictability (e.g., *“My family moves frequently”, “I or my partner was unpredictable”*), responding with 1 - “Yes” or 0 - “No”. Items on the QUIC were summed such that higher scores indicated greater unpredictability (mean = 1.77, sd = 2.03). These items exhibited acceptable internal consistency (α = .68). For a full list of items on the Family Routines and QUIC, see Supplementary Table S1.

### Analytic strategy

#### Aim 1: Characterize unpredictability in economic hardship

Our first aim was to compute and characterize economic unpredictability and explore descriptive relations. This required a series of iterative steps outlined below.

##### Compute unpredictability statistics

For each individual time series of economic hardship, four unpredictability statistics were computed in R version 4.2.3. Coefficient of variation and noise were calculated using the *stats* package (R Core Team, 2013). Changepoints in mean and variance were calculated using the *changepoint* package (Killick & Eckley, 2014).

These derived unpredictability statistics were then used to conduct a PCA (PCA; Greenacre et al., 2022; Wold et al., 1987) using the *psych* R package (Revelle & Revelle, 2015). This method afforded a systematic reduction in data dimensionality, enabling us to identify the core aspects of our construct of economic unpredictability. PCA does this by transforming each of the unpredictability indices into a new set of uncorrelated components, thereby streamlining subsequent analysis conducted in our second aim (see below) without significant loss of information. To conduct the PCA, we first standardized the unpredictability indices to have a mean of zero and a variance of one. Subsequently, we computed the covariance matrix to understand the relations between variables. Eigenvalues and eigenvectors were then derived from this matrix, leading to the selection of principal components that captured the most variance in the data.

##### Descriptive correlations and group comparisons

To explore average associations between the individual unpredictability statistics, our PCA-derived components of economic unpredictability, and questionnaire-based measures of unpredictability, we examined pairwise correlations between the variables. We also explored whether these variables differed between sociodemographic groups. To do this, we generated descriptive statistics and plots of the unpredictability indices separated by group to examine descriptive (i.e., non-inferential) comparisons and characterize patterns.

#### Aim 2: Determine the effect of economic hardship and unpredictability on children’s self-regulation

Our second aim was to isolate the relation between overall economic hardship (i.e., hardship severity indexed as the mean across time) and our derived unpredictability principal component (from our first aim) on children’s self-regulation. To do this, we applied Inverse Probability of Treatment Weighting (IPTW; Austin et al., 2015), an approach that enhances causal inference in observational data by balancing on potential confounds across all levels of an environmental exposure (Austin et al., 2015).

Importantly, IPTW overcomes limitations of traditional regression based techniques that are particularly sensitive to specific combinations of conditioning covariates. In regression models, statistical adjustment for many confounding variables can lead to overly complex models, collinearity among variables, and/or strategic inclusion of only confounding variables that result in desirable effects (Rubin, 2001). Rather than controlling for shared variation between an environmental exposure and confounds, IPTW generates a pseudo-population whereby the relationship between confounders and the exposure becomes orthogonal, or uncorrelated. This quasi-experimental technique aligns with the principles of randomized experimentation by statistically balancing confounders across exposure levels, thereby mimicking random assignment. Consequently, in this pseudo-population, any differences in our self-regulation outcome across levels of our exposure (economic hardship and unpredictability) are attributed to the exposure itself, assuming that all relevant confounders are accounted for and appropriately balanced.

The covariates included in our balancing models were selected based on their theoretical and empirical relevance as potential confounders influencing both economic hardship/unpredictability and child self-regulation. We also sought to maintain parsimony to address the constraints of our small sample size. Parsimony was critical to avoid overfitting, inflated variance in the estimated weights, and a reduced effective sample size (i.e., the weighted sample size after accounting for variability introduced by the weights), which could compromise statistical power. To strike this balance, we included parent education and family income as proxies for SES and access to resources, given their role in shaping exposure to economic hardship/unpredictability and the developmental environment that supports self-regulation. Race/ethnicity was included to capture structural inequities that influence exposure to economic hardship and can affect child outcomes through systemic barriers or supports. Child age was included to account for developmental differences that influence self-regulation and the duration of exposure to economic hardship/unpredictability. Finally, child disabilities and health were included for their potential impact on family economic conditions, such as increased medical costs or caregiving demands, as well as their contribution to stress pathways that affect self-regulation. A total of 15 confounds were used in the balancing models, taking into account dummy-coded categorical confounds (which were all variables except for age and child health).

We used the *WeightIt* R package (Griefer, 2019) to create balancing weights for each environmental exposure (economic hardship and each unpredictability component derived from aim 1), and to test a series of weighting approaches for continuous exposures.The optimal weighting method was identified by evaluating balance output across approaches. Specifically, we examined the postbalance associations between confounders and exposure; correlations of less than .10 are considered reasonably balanced (Stuart, 2008). The resulting propensity score weights, representing the weighted contributions of the confounding variables, were subsequently applied in weighted regression models. Separate models were fitted for each environmental exposure to examine their unique associations with children’s self-regulation. As the unpredictability indices and mean hardship variables are on different scales, the parameter estimates cannot be compared directly. Thus, we standardized effect sizes, in lieu of inferential comparisons.

#### Openness and transparency

Despite being exploratory with respect to theory, we pre-registered our study methods and statistical approach on the Open Science Framework (OSF). Code and supplementary materials, as well as the pre-registration, can be found on the OSF page here: https://osf.io/vzr2y/?view_only=ff0842694c1744c6bf9cb71d41b1c903. We deviated from our pre-registration in three ways. First, although we planned to calculate six unpredictability statistics in aim 1, we were only able to calculate four given unanticipated constraints with the data that deemed the adequate calculation of entropy and autocorrelation unfeasible. Specifically, as mentioned in the pre-registration, these two statistics require the time series to have close to equal spacing between time points, which the current data did not meet. Second, we were unable to use weighted path analysis for aim 2 as pre-registered given that our analytic sample was smaller than anticipated; thus we used weighted linear regression. Third, for aim 2, we added analyses in addition to the two pre-registered models (see below). Fourth, during the revision process, we identified and corrected a coding error in the computation of the noise variable. We transparently describe this correction in the supplement (see Figure S2) and reran all relevant analyses accordingly.

## Results

### Aim 1: Characterize unpredictability in economic hardship

#### Deriving environmental statistics and dimensions

**Figure 1.**
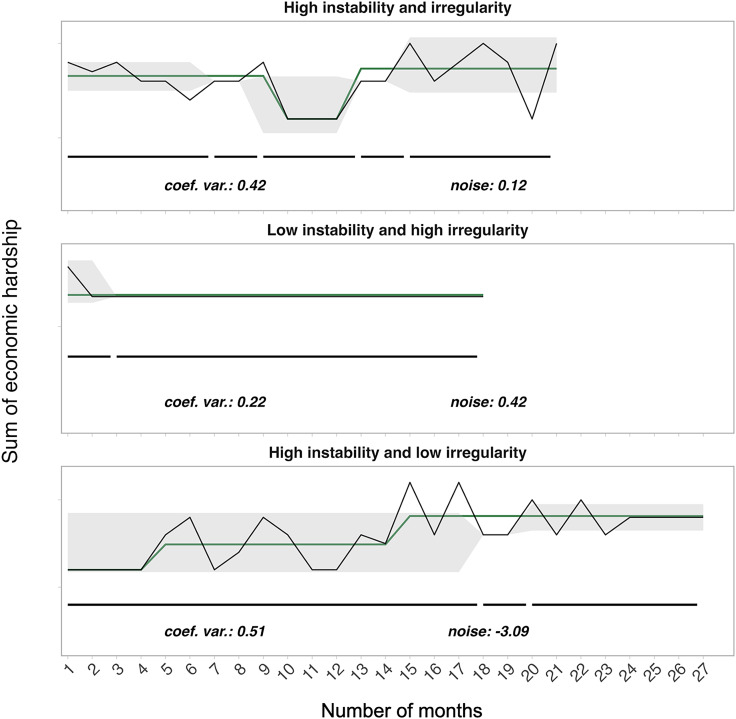
Trajectories of economic hardship illustrating possible combinations of unpredictability indices sampled from three participants. Each row depicts a participant’s unique time series of caregiver-reported economic hardship (y-axis) across the number of months (x-axis) for which a response was provided. ‘Instability’ refers to unpredictability indices capturing the amount (frequency and magnitude) of changes in economic hardship (coefficient of variation, changepoints in mean and variance); ‘Irregularity’ refers to the noise index, which captures the patterns of irregularity in the time series after accounting for systematic change. The green line tracks changes in mean and the gray shading tracks changes in variance in monthly material hardship levels. Black horizontal lines below the time series track segments of stable variance, with breaks denoting where a changepoint occurs. Individual values for coefficient of variation and noise (raw value) are displayed at the bottom of each plot. Note that participant-level time series ranged from a minimum of 15 months to 36 months.

Figure 1 depicts the four economic unpredictability statistics – the coefficient of variation, changepoints in mean, changepoints in variance, and noise – calculated for each individual family. Coefficient of variation and changepoints in mean and variance were moderately positively correlated (*rs* = 0.28 to 0.65). Noise was moderately to strongly positively correlated with coefficient of variation and changepoints (*rs* = 0.53 to 0.80). Pairwise correlations can be found in Supplemental Figure S1.

**Figure 2.**
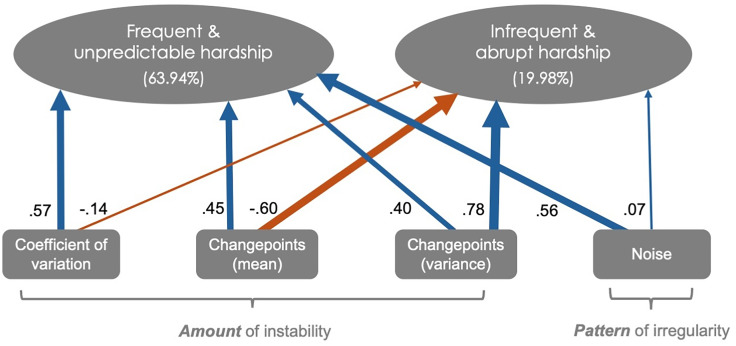
Principal component analysis on four standardized environmental statistics representing aspects of economic unpredictability. Circles represent the two components that explained the most variance (84%) and squares represent each environmental statistic. The width of the arrows denote the absolute magnitude of the loading for each statistic on each component. Blue and orange denote positive and negative loadings, respectively. PC1 is deemed to represent frequent and unpredictable fluctuations in economic hardship, characterized by high positive loadings across all indices. PC2 is deemed to represent infrequent but abrupt fluctuations in economic hardship, characterized by a contrast between changepoint variance and changepoint mean, with weaker contributions from the coefficient of variation and noise.

To further explore the dimensionality of economic unpredictability, a PCA was conducted on the four indicators, which were standardized to place them on the same scale. The PCA identified two principal components that together explained 83.92% of the total variance, with PC1 accounting for 63.94% and PC2 for 19.98% (Figure 2).

The first principal component (PC1), which we deem to represent frequent and unpredictable hardship, is characterized by high positive loadings for the coefficient of variation (loading = 0.57), changepoints in mean (loading = 0.45), changepoints in variance (loading = 0.40), and noise (loading = 0.56). This component captures variation and frequency of volatile changes in environmental conditions (via high coefficient of variation and changepoints), whereby such patterns in variation are unpredictable (via high noise).

The second principal component (PC2), which we deem to represent infrequent but abrupt hardship, reflects a contrast between changepoints in variance (loading = 0.78) and changepoints in mean (loading = -0.60), with weaker contributions from the coefficient of variation (loading = -0.14) and noise (loading = 0.07). This component captures environments where change in economic conditions are highly variable in timing (via high changepoints in variance),occur less frequently (via low changepoints in mean), and follow a sporadic pattern (via minimal influence from noise and coefficient of variation). To create a single index of economic unpredictability, we calculated a weighted average of PC1 and PC2, using their respective proportions of variance explained as weights (PC1 = 63.94%, PC2 = 19.98%). This approach was chosen to retain the contribution of both principal components, with PC1 capturing systematic instability and PC2 representing dynamic variability. By weighting the components based on their explanatory power, this composite index prioritizes the dominant dimension of unpredictability (PC1) while still integrating the meaningful variance captured by PC2. This method ensures a balanced representation of the underlying dimensions while simplifying subsequent analyses (aim 2).

#### Descriptive relations between economic unpredictability indices, self-report measures, and sociodemographics

**Figure 3.**
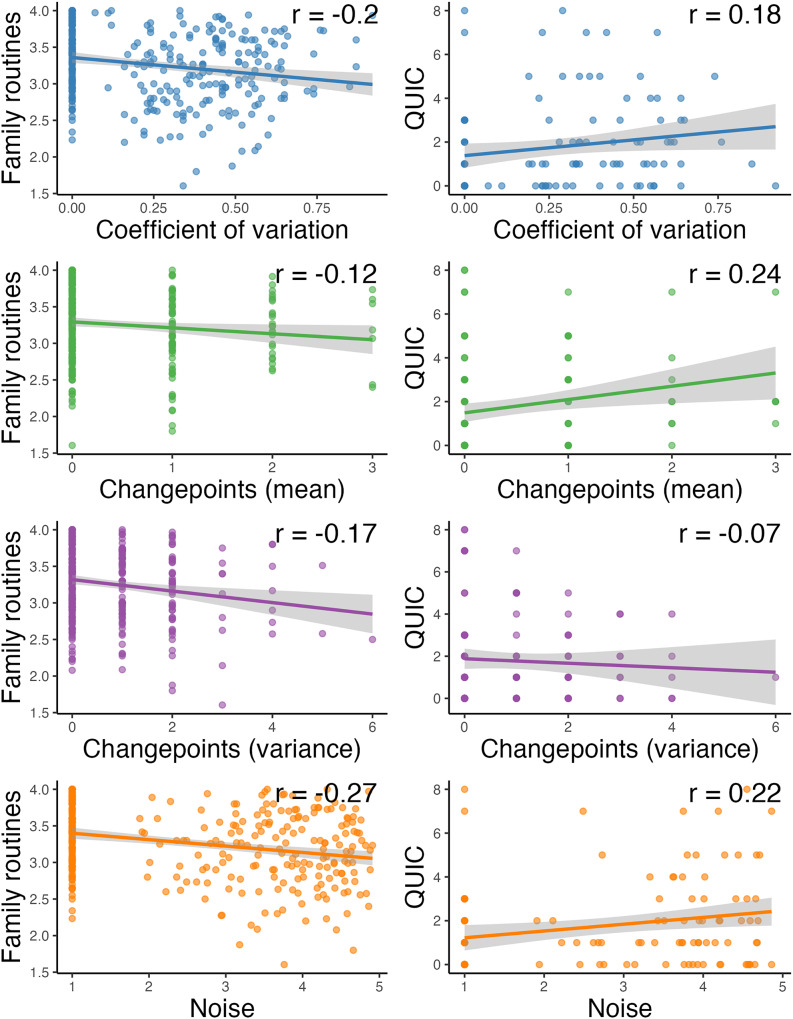
Relations between each unpredictability statistic (x-axis, ed by statistic) and family routines (left column) and the Questionnaire of Unpredictability in Early Childhood (right column). Gray shading depicts 95% confidence intervals.

Associations between environmental indices and questionnaire-based measures of unpredictability can be found in Figure 3. All unpredictability indices were weakly negatively correlated (*r*s = −0.12 to −0.27) with caregiver-reported family routines. For caregiver-reported environmental unpredictability assessed via the QUIC, coefficient of variation, changepoints in mean, and noise were weakly positively correlated (*rs* = 0.18–0.22). In contrast, changepoints in variance was weakly negatively correlated with caregiver-reported unpredictability (*r* = -0.07).

**Figure 4.**
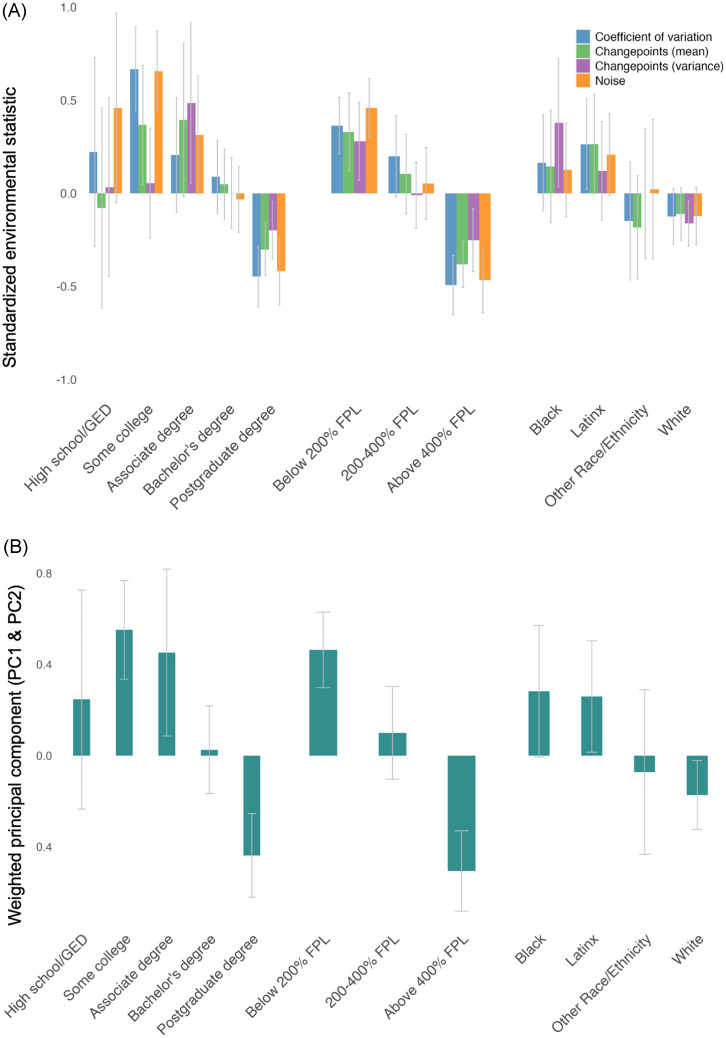
Panel A denotes descriptive comparisons of individual environmental statistics across sociodemographic groups. Panel B denotes the weighted combination of the two economic unpredictability components derived from principal component analysis across sociodemographic groups. Bars indicate group-level averages ed by environmental statistic and error bars indicate 95% confidence intervals. GED = General Education Development; FPL = Federal Poverty Level.

Descriptive (non-inferential) comparisons between economic unpredictability statistics (individual indices and weighted principal component index) and sociodemographic factors are illustrated in Figure 4. Here we additionally report means and standard deviations for the weighted principal component index. Regarding parent education, households with some college or an associate degree exhibited the highest economic unpredictability (mean = 0.55, sd = 0.76; mean = 0.45, sd = 1.02), while those with a high school diploma/GED or bachelor’s degree showed similarly lower levels (mean = 0.25, sd = 0.92; mean = 0.03, sd = 1.02). Postgraduate households demonstrated low economic unpredictability (mean = -0.44, sd = 1.00). For household income, unpredictability was highest among households at or below 200% of the federal poverty threshold (mean = 0.46, sd = 0.86), moderate for those at 200–400% (mean = 0.10, sd = 1.04), and lowest for those at or above 400% (mean = -0.51, sd = 0.97). Racial/ethnic comparisons showed the highest unpredictability among Black (mean = 0.28, sd = 1.06) and Latinx (mean = 0.26, sd = 1.01) families, while White families (mean = -0.17, sd = 1.01) and other racial groups (mean = -0.07, sd = 1.04) demonstrated lower economic unpredictability.

### Aim 2: Impact of economic unpredictability and mean hardship on children’s self-regulation

Economic unpredictability and mean hardship was moderately positively correlated (*r* = .50), suggesting economic unpredictability is a related yet distinct exposure. Weighted linear regression models were used to isolate the unique impact of each environmental exposure on children’s self-regulation challenges.

#### Economic unpredictability model

Examination of initial imbalance revealed that confounders were minimally to moderately correlated with the unpredictability principal component index (|r| = 0.02–0.37). The Bart weighting method achieved the best balance, successfully balancing 15 of the 15 confounders. Among the balanced confounders, the maximum absolute correlation with the exposure was only −0.09. The effective sample size was reduced to 95 (from 125 unweighted), reflecting the expected trade-off of reduced precision for improved balance.

**Figure 5.**
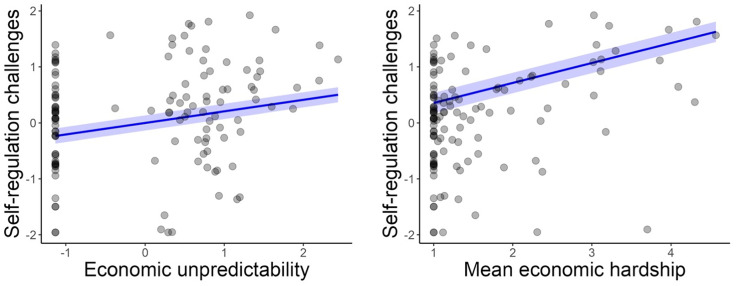
Relations between economic unpredictability (left panel) and mean economic hardship (right panel) and child self-regulation challenges from weighted regression models. The blue line indicates the predicted relation superimposed on the raw data points.

In the weighted regression model (Figure 5), greater economic unpredictability was significantly associated with increased self-regulation challenges (*β* = 0.25; *B* = 0.21, 95% CI [0.07, 0.34], *p* = .005). These findings suggest that children exposed to higher levels of economic unpredictability may have slightly more challenges in self-regulation.

#### Mean economic hardship model

Examination of initial imbalance for the mean economic hardship models indicated that confounders were also minimally to moderately correlated with the exposure (|r| = 0.03–0.38). The Covariate Balancing Propensity Score method provided the best balance, successfully balancing 14 of the 15 confounders. The remaining imbalance was observed in the 200% – 400% poverty threshold category, which retained a marginal correlation with the exposure of -0.11, slightly exceeding the threshold of 0.10. Among the balanced confounders, the maximum absolute correlation with the exposure was 0.10. The effective sample size was reduced to 104 (from 125 unweighted), again illustrating the trade-off between precision and achieving well-balanced confounders.

In the weighted regression model (Figure 5), higher levels of mean economic hardship were significantly associated with greater self-regulation challenges (*β* = 0.35; *B* = 0.36, 95% CI [0.17, 0.54], *p* < 0.001). This suggests that children exposed to higher average economic hardship have more challenges in self-regulation, emphasizing the cumulative impact of sustained economic challenges on self-regulation.

### Non-preregistered analyses

Non-preregistered models examined the relation between specific unpredictability statistics and self-regulation challenges. The coefficient of variation showed a nonsignificant positive association with self-regulation challenges (*β* = 0.14; *B* = 0.50, 95% CI [−0.07, 1.06], *p* = 0.08). Changepoints in mean showed a significant positive association (*β* = 0.22; *B* = 0.23, 95% CI [0.10, 0.36], *p* = 0.01), as did changepoint in variance (*β* = 0.30; *B* = 0.23, 95% CI [0.12, 0.35], *p* < 0.001). Noise showed a nonsignificant positive association with self-regulation challenges (*β* = 0.11; *B* = 0.07, 95% CI [-0.05, 0.18], *p* = 0.248). These results suggest that specific aspects of unpredictability, particularly those related to the amount of instability (e.g., changepoints), may be related to more self-regulation challenges in early childhood.

## Discussion

Extensive research has established that early exposure to economic hardship is linked to children’s self-regulation development (e.g., Blair & Raver, 2012). However, far less is understood about the natural ecology of how hardship fluctuates over time and whether those fluctuations follow discernable patterns that may exert unique demands on children’s self-regulation development. Notably, few studies have been methodologically equipped to quantify and isolate these effects. The present study addresses this gap by leveraging contemporary theoretical frameworks and quasi-experimental methods within a socioeconomically diverse sample, tracking over 15 monthly assessments of economic hardship. Our findings suggest that on average, economic unpredictability involves frequent fluctuations in hardship that follow an unstructured, random pattern. However, at least in the current sample, economic unpredictability is not created equal across families – it is disproportionately experienced by those from racially minoritized and lower-SES backgrounds, reflective of historical inequities. Finally, while both overall economic hardship and unpredictability were associated with greater self-regulation challenges in early childhood, the effect of hardship severity was qualitatively stronger. These findings advance the rapidly evolving field of environmental unpredictability and highlight the need to consider temporal patterns in economic stressors, alongside mean levels, in both research and policy.

### Characterizing economic unpredictability

Drawing on a recently developed environmental statistics framework (Walasek et al., 2024), we generated four indices of economic unpredictability to characterize how subjective economic hardship (i.e., ability to meet basic needs) fluctuates over time for families with young children. Three of these indices – changepoints in mean, changepoints in variance, and coefficient of variation – quantified the *amount* (i.e., frequency and magnitude) of instability, measuring how often hardship levels shifted and how extreme those shifts were. For example, a family experiencing multiple changepoints in their mean level of hardship may have undergone several significant economic transitions, such as job loss, a shift to public assistance, or eviction. Similarly, high changepoints in variance indicate periods of relative financial stability punctuated by sudden hardship shifts, such as unexpected medical expenses or an abrupt reduction in work hours. A high coefficient of variation would indicate that a family’s economic hardship fluctuated dramatically relative to their average level of hardship. Together, these indices – which were moderately to highly positively correlated with each other – captured key aspects in the amount of systematic shifts in economic hardship over time.

The fourth index, noise, provided a different lens on economic unpredictability by capturing the temporal *pattern* of hardship fluctuations. Higher noise values indicate greater unpredictability, where hardship levels change in irregular ways. In contrast, lower noise values reflect more predictable fluctuations, such as regularly recurring economic strain before payday or seasonal income cycles. This index was moderately to strongly positively correlated with the other three unpredictability indices. In other words, families experiencing greater unpredictability in terms of coefficient of variation and changepoints in mean and variance also showed greater unpredictability in noise, on average.

To better understand average associations between these indices, we modeled their underlying structure using PCA. Rather than reflecting a single, uniform construct, our analysis revealed two distinct dimensions of economic unpredictability, suggesting that families experience different types of hardship, each with unique patterns and implications. The first dimension, *frequent and unpredictable hardship*, accounted for 64% of the variance, meaning that it represents the dominant pattern of economic unpredictability in our sample. This dimension was characterized by high positive loadings for changepoints in mean, changepoints in variance, coefficient of variation, and noise. This pattern reflects frequent and substantial shifts in economic hardship that occur in irregular and unpredictable ways. Families experiencing this form of hardship may contend with sudden income drops, shifting access to resources, or inconsistent expenses – forms of instability that are both high in magnitude and difficult to anticipate. Such environments offer little space to plan or buffer against shocks, potentially increasing stress and caregiving challenges (Li & Belsky, 2022; Li et al., 2018).

The second dimension, *infrequent and abrupt hardship*, accounted for 20% of the variance, capturing an additional but distinct form of economic unpredictability. Unlike *frequent unpredictable hardship*, this dimension was characterized by a high positive loading for changepoints in variance, a strong negative loading for changepoints in mean, with minimal contributions from coefficient of variation and noise. This combination suggests that some families experienced fewer frequent shifts in hardship levels but, when these shifts did occur, they were abrupt and substantial. In other words, these families had extended periods of economic stability followed by sudden, large changes in hardship (e.g., health bills, job loss, change in benefits or household composition).

These findings build on prior research on economic unpredictability, which has largely relied on measures of the magnitude of instability, such as the coefficient of variation (e.g., Liu et al., 2023) or a sum of transitions in and out of poverty (e.g., Raver et al., 2013). A limitation of most of these previously used measures is that they aggregate across time points rather than capturing how economic conditions change dynamically. Our indices explicitly modeled these dynamics, relying on the order of repeated assessments of economic hardship to capture when and how families’ ability to meet their basic needs shifted. Additionally, by incorporating noise as an index of temporal structure, we moved beyond measuring the amount of instability to characterizing the degree of regularity in economic fluctuations after accounting for systematic shifts in mean and variance. By integrating multiple indices that reflect distinct but interrelated features of economic unpredictability, our study provides a more nuanced and comprehensive approach for understanding how economic unpredictability unfolds in families’ lives.

### Relations between economic unpredictability indices and questionnaire-based measures of environmental unpredictability

After conducting the analyses reported thus far, we further sought to address how economic unpredictability translates into families’ lived experiences of daily stability and routine. Specifically, do families who experience greater unpredictability in their economic circumstances also perceive their everyday lives as more unstable? To examine this, we explored associations between our four economic unpredictability indices and caregiver-reported measures of family routines (e.g., regular playtimes and mealtimes) and general environmental unpredictability (e.g., caregiver stability, residential moves, and fluctuations in childcare arrangements).

Across both self-report measures, families experiencing more frequent and extreme economic hardship fluctuations, in particular higher coefficient of variation and changepoints in mean, tended to report slightly less stable daily environments. This suggests that certain aspects of economic instability may have spillover effects on caregiving structure and household organization. When considering the pattern of this instability, as indexed by noise, more erratic shifts in economic conditions were associated with greater unpredictability in daily home life. However, all of these associations were small, indicating that many families are able to maintain structured routines despite economic strain. Predictable daily structures, such as regular mealtimes and bedtime routines, may provide children with stability even when economic conditions fluctuate unpredictably. This aligns with prior work suggesting that routines serve as an important buffer for children’s development, particularly in contexts of adversity (see Selman & Dilworth-Bart, 2024 for a meta-analysis).

Taken together, these findings indicate that our derived indices of economic unpredictability do not map cleanly onto families’ perceptions of instability in their daily lives. While economic hardship can introduce stress and uncertainty, families may develop adaptive strategies to maintain a sense of predictability in their home environments. It is important to note that our analysis focuses solely on unpredictability in economic resources, and other domains – such as parental mood fluctuations or caregiving instability – may show different patterns (e.g., Doom et al., 2024; Ugarte & Hastings, 2024).

### Racial and socioeconomic inequities in exposure to economic unpredictability

While families may find ways to buffer children from daily unpredictability, structural inequities mean that some groups face greater and more persistent economic instability, leaving different opportunities for coping. Thus, in addition to identifying broad patterns of economic unpredictability and its connections to daily life, we examined which families were disproportionately affected by these experiences. To do so, we explored differences across racial and socioeconomic groups, using both individual unpredictability indices and a composite index that captured a weighted combination of the two dimensions from our PCA.

Families with the highest levels of economic unpredictability included those with lower education (i.e., some college or an associate degree), incomes below 200% of the federal poverty level (FPL), and Black and Latinx families. At the individual index level, these groups exhibited moderately high coefficients of variation and changepoints in mean and variance, combined with high noise. This suggests that their experiences of economic hardship involved frequent and substantial shifts, in a way that followed an unsystematic pattern. By contrast, families with the lowest levels of economic unpredictability – those with postgraduate degrees, incomes above 400% federal poverty level, and White families – tended to experience steadier economic conditions. These groups had the lowest coefficient of variation and changepoints in mean and variance, as well as noise, indicating that when economic hardship did occur (if at all), it was potentially more anticipated and systematic in nature.

These patterns observed in our sample highlight how families’ economic conditions are shaped by broader structural inequities, with lower-income and racially minoritized families experiencing hardship that is both frequent and marked by unpredictable fluctuations. A family living below the poverty line, for example, may be in a precarious position where even small fluctuations in income – such as a reduction in work hours, inconsistent gig earnings, or delays in government assistance – can directly affect their ability to meet basic needs. For many, economic hardship is not only persistent but also interconnected with other forms of instability, such as housing precarity (Kull et al., 2016), fluctuating access to childcare (Luhr et al., 2022), and multigenerational financial obligations (Keene & Batson, 2010). Because their income is so closely tethered to their ability to meet basic needs, even minor unexpected expenses can lead to repeated periods of heightened hardship, often with little warning or consistency in timing. In contrast, a highly affluent family is far less likely to experience these kinds of frequent and unpredictable fluctuations in subjective hardship. With steady incomes, intergenerational wealth, safety nets, and access to financial buffers such as savings accounts or loans, economic instability – if it arises – is less likely to translate into tangible disruptions in meeting basic needs. These families may go long stretches reporting no hardship at all, and when they do encounter economic strain, it tends to follow predictable patterns such as planned financial transitions or seasonal expenses (e.g., family vacations, holiday expenses).

### Economic hardship severity versus unpredictability: related but distinct predictors of children’s self-regulation

Our final aim sought to disentangle the effects of economic unpredictability from overall hardship severity on children’s self-regulation. We found that economic unpredictability and overall hardship were only moderately correlated (*r* = .50), reinforcing prior research that these are related but distinct experiences (Miller et al., 2025; Liu et al., 2022; DeJoseph et al., 2021). While overall hardship represents the severity of economic strain, unpredictability captures dynamic shifts in economic conditions, which may uniquely shape children’s regulatory capacities (McLaughlin et al., 2014; Ellis et al., 2022; Frankenhuis et al., 2020).

Consistent with prior research, higher overall economic hardship predicted greater challenges in children’s self-regulation. This finding aligns with extensive evidence linking poverty to self-regulatory challenges in early childhood (Palacios-Barrios & Hanson, 2019; Raver et al., 2013). Economic unpredictability was also associated with greater self-regulation challenges, but the effect was qualitatively weaker than that of hardship severity. Recent research by Li and Belsky (2022) corroborates this finding, showing that greater income harshness (i.e., overall less income over time) was associated with greater internalizing and externalizing behaviors among kindergarten children, whereas income unpredictability showed a relatively weaker effect on externalizing and did not predict internalizing.

A unique contribution of our study compared to prior work is our ability to explore whether and which features of unpredictability matters most for predicting individual differences in children’s self-regulation. Our non-preregistered analyses revealed that changepoints in variance emerged as the strongest predictor of self-regulation challenges, followed by changepoints in mean. Coefficient of variation and noise showed small positive associations with self-regulation, but these were not statistically significant. The strong role of changepoints in variance and mean suggests that long periods of stability punctuated by abrupt economic shocks may be particularly important for shaping children’s emerging self-regulation skills. These sudden shifts may create stressful, destabilizing experiences that disrupt children’s ability to develop consistent regulatory strategies. Why might abrupt economic shocks be so disruptive? One possibility is that caregivers navigating sudden economic instability struggle to maintain predictable caregiving behaviors, emotional availability, and consistent expectations for their children. Fluctuations in resources may create instability in parental stress, mood, and responsiveness – factors that shape children’s self-regulation (Ugarte & Hastings, 2024). Prior research has shown that unpredictable maternal behaviors, such as inconsistent vocalizations, facial expressions, and physical touch, are predictors of cognitive and emotional challenges in infants and young children (Davis et al., 2017; Forest et al., 2025). Economic instability may disrupt caregiving in similar ways, introducing unpredictable patterns in parental attention and support that make it harder for children to regulate emotions and behavior.

It is important to note that in our analyses, all standardized effect sizes for the composite measure of economic unpredictability and individual indices were qualitatively smaller than the effect of overall hardship severity. This may be because unpredictability shapes both enhancements and impairments in self-regulation, a distinction we were unable to disentangle in the current study. Prior research suggests that unpredictable environments can enhance attention shifting and working memory updating – skills that support adaptation to rapidly changing conditions – albeit at the cost of impairing inhibitory control (e.g., Fields et al., 2021; Young et al., 2018). In some cases, these enhancements are observed only under experimental conditions that explicitly direct attention to environmental unpredictability (e.g., through priming). This highlights the context-dependent nature of these effects and underscores the need for future work to disentangle when and for whom such adaptations emerge. Additionally, differences in how unpredictability has been measured across studies complicate direct comparisons, underscoring the importance of using consistent frameworks for operationalizing instability. As the field of environmental unpredictability research continues to expand, leveraging formal statistical approaches like those used in the present study will be critical for building more comprehensive models of how unpredictability shapes self-regulation across development. Additionally, assessing how different aspects of economic hardship unfold across time, space, and subjective perceptions from caregivers and children will provide a more nuanced and comprehensive understanding of these processes (Munakata et al., 2023; Walasek et al., 2024).

### Strengths, limitations, and future directions

This study is the first to formally quantify several economic unpredictability indices across a relatively large sample, with participants contributing between 15 and 36 time points – exceeding the six or fewer time points typically used in prior research. Additionally, our sample was diverse in terms of race, income, and education, and data collection took place during a historically unstable economic period (the COVID-19 pandemic), enhancing the relevance of our findings to families navigating economic uncertainty. Our use of rigorous measurement and quasi-experimental methods further strengthens the validity of our approach.

Despite these contributions, our study has several limitations. First, while in some ways a strength, the pandemic context may limit the generalizability of our findings. COVID-19 introduced widespread disruptions in employment, childcare access, and public assistance systems that may have amplified the frequency and severity of economic shocks, particularly for families already facing structural disadvantage. Although unpredictability in economic resources is a persistent challenge for many families, it remains an open question whether the specific patterns and associations observed here would emerge under more typical economic conditions. Future work is needed to replicate these findings in more stable periods across different populations and to examine how macroeconomic context interacts with family-level unpredictability.

Second, we were unable to calculate additional unpredictability indices such as entropy and autocorrelation due to the unequal spacing of survey responses, which prevented meaningful interpretations of time lags. This also limited our ability to examine the precise timing of hardship fluctuations. While our study leveraged month-to-month variation in hardship, future work should explore even finer-grained variation within shorter time frames (e.g., weekly or daily fluctuations). Prior research has shown that access to resources and parenting practices may differ at the beginning versus the end of the month (e.g., Ellwood-Lowe et al., 2022), highlighting the potential importance of capturing within-month variation and its cascading effects on child outcomes. Additionally, our first aim was limited to descriptive analyses, providing an important first step in characterizing patterns of economic unpredictability and building greater conceptual clarity in this rapidly growing area of research. Future work should report similar descriptives while incorporating inferential comparisons across groups and measures to further clarify how different forms of unpredictability manifest across different families.

This study also raises several open questions for future research on the measurement of environmental unpredictability. The environmental statistics framework we adopted (Walasek et al., 2024) provides a standardized approach to capturing economic unpredictability, but an important next step is determining whether similar dimensions (i.e., frequent unpredictable hardship and infrequent abrupt hardship captured in our PCA) emerge in other domains of environmental unpredictability. For instance, do families experience comparable patterns of unpredictability in neighborhood violence exposure (Farkas & Jacquet, 2024; Walasek et al., 2024), caregiving behaviors (see Ugarte & Hastings, 2024 for a review), parental mood fluctuations, resource access, or housing instability? Additionally, future research should work toward bridging levels of analysis by examining how macro-level factors – such as policy-driven income supports, labor market precarity, and safety net programs – shape the micro-level experience of economic unpredictability. Understanding whether patterns of unpredictability at one level align with those at another, and whether objective and subjective indicators of unpredictability converge, will be critical for advancing both research and policy.

With respect to child outcomes, our broad measure of self-regulation, combined with the smaller sample size available for this aim, limited our ability to conduct fine-grained analyses on potential patterns of developmental adaptation (i.e., combinations of enhancements and impairments). As mentioned above, it will be important for future work to map different dimensions of environmental unpredictability onto ways in which children’s self-regulation may be enhanced, impaired, or left intact, as well as ways in which self-regulation strategies are adaptively tailored to environmental or contextual demands. A principled exploration is needed, alongside confirmatory hypothesis testing, to uncover the conditions under which certain cognitive and regulatory skills may develop (mal)adaptively in response to specific unpredictable environments (Young et al., 2024).

Lastly, our study relied on subjective caregiver-reported measures of economic hardship and child self-regulation, which in some ways is a strength (e.g., shared reporter variance, subjective perceptions of hardship), introduces potential biases. For example, caregivers who perceive their economic circumstances as more unstable may also be more likely to view their children as having greater self-regulation challenges, and genetic factors could further contribute to both economic instability and self-regulation. Future research could leverage genetically informed or multi-informant designs to better disentangle these influences and reduce potential confounding. Another issue related to relying on caregiver reports is measurement invariance. The way caregivers perceive and report on these constructs may vary based on cultural background, economic context, or psychological factors, making it essential to test and adjust for non-invariance when comparing different groups (DeJoseph et al., 2021, 2022). While our sample size was too small to formally test for measurement invariance, this remains an important avenue for future research, particularly as efforts to measure unpredictability across multiple domains and populations continue to expand. Relatedly, we relied on a cumulative count index of economic hardship, which provided a broad proxy for families’ subjective experiences of strain but may not fully capture the diverse and nuanced ways families experience economic hardship. Future work would benefit from incorporating more comprehensive, multidimensional measures of economic conditions to build on and refine these approaches.

## Conclusion

Our findings reveal that economic unpredictability is not a monolithic experience – some families navigate frequent and unpredictable fluctuations in hardship, while others experience less frequent changes that vary in timing and can still pose significant challenges. Families from racially minoritized and lower-income backgrounds disproportionately bear the burden of these recurring fluctuations, reflecting deeply rooted structural inequities. For caregivers already stretched thin, the constant uncertainty of whether they will be able to meet their family’s basic needs can be exhausting, leaving little room to plan, save, or create a stable environment for their children. While both economic unpredictability and overall hardship were linked to self-regulation challenges in early childhood, hardship severity had a stronger effect, underscoring the potential toll of sustained economic strain on children’s ability to regulate emotions and behavior. These findings highlight the urgent need for policies that do more than provide temporary relief – they must promote lasting stability. Expanding predictable income supports (e.g., guaranteed basic income programs), strengthening housing and childcare assistance, and reforming public benefits to prevent abrupt disruptions in household resources could ease the economic unpredictability so many families face. Capturing these dynamic patterns of economic resources is a critical step toward designing policies and programs that reflect the realities of economic instability and create pathways toward greater security.

## Supporting information

10.1017/S0954579425100771.sm001 DeJoseph et al. supplementary material DeJoseph et al. supplementary material

## Data Availability

The data that support the findings of this study are not publicly available.

## Supplementary material

The supplementary material for this article can be found at https://doi.org/10.1017/S0954579425100771.

## Pre-registration statement

This study was pre-registered on the Open Science Framework. The pre-registration, along with analysis code and supplementary materials, is available at: https://osf.io/vzr2y/?view_only=ff0842694c1744c6bf9cb71d41b1c903.
