# Supplementary material for: Hidden dynamics of economic hardship: Characterizing economic unpredictability and its role on self-regulation in early childhood
Source: Dev Psychopathol. 2025 Oct 29:1–16. Online ahead of print. doi: 10.1017/S0954579425100771 (PMC12773789; doi:10.1017/S0954579425100771)
Supplement: DeJoseph et al. supplementary material [file S0954579425100771sup001.pdf]

Supplemental Materials for Hidden dynamics of economic hardship: Characterizing economic unpredictability and its role on self-regulation in early childhood

Table S1
Items on questionnaire-based measures of unpredictability.

	Items	Scoring
Family Routines (developed by RAPID team)<br><br>**Prompt:** Was this a routine prior to the COVID-19 pandemic?/Has this been a routine since the beginning of the COVID-19 pandemic?	Working parent(s) have a regular play time with the children after work	1 - Almost every day<br>2 - 3-5 Times a week<br>3 - 1-2 Times a week<br>4 - Almost never
	Children did the same things each morning as soon as they woke up	
	Family had certain 'family time' each week when they did things together at home	
	Children went to bed at the same time almost every night	
	Whole family ate dinner together almost every night	
Questionnaire of Unpredictability in Childhood (QUIC; Glynn et al., 2019)	There are often people coming and going in my house that my child(ren) did not expect to be there	0 - No<br>1 - Yes
	My family moves frequently	
	My child(ren) changed preschools or childcare centers/programs they attend frequently, or changed mid-year	
	I and my child(ren) live in a cluttered house (e.g., piles of stuff everywhere)	
	There was a period of time when I and my child(ren) did not feel safe in my home	
	I or my partner were often late to pick my child(ren) up (e.g., from school, daycare)	
	My child(ren) usually knew when I and/or my partner were going to be home	0 - Yes<br>1 - No
	I or my partner have punishments towards my child(ren) that were unpredictable	0 - No<br>1 - Yes
	I or my partner would plan something for the family but then not follow through with the plan	

	I or my partner was organized	0 - Yes<br>1 - No
	I or my partner was unpredictable	
	I or my partner could go from calm to furious or stressed/nervous in an instant	
	There was a long period of time when my child(ren) didn't see my or my partner (e.g., military deployment, jail time, custody arrangements)	
	My child(ren) experienced changes in their custody arrangement	
	I or my partner changed jobs frequently; or there were times when I or my partner were unemployed and couldn't find a job	0 - No<br>1 - Yes
	I and my partner had a stable relationship with each other	
	I and my partner got divorced	

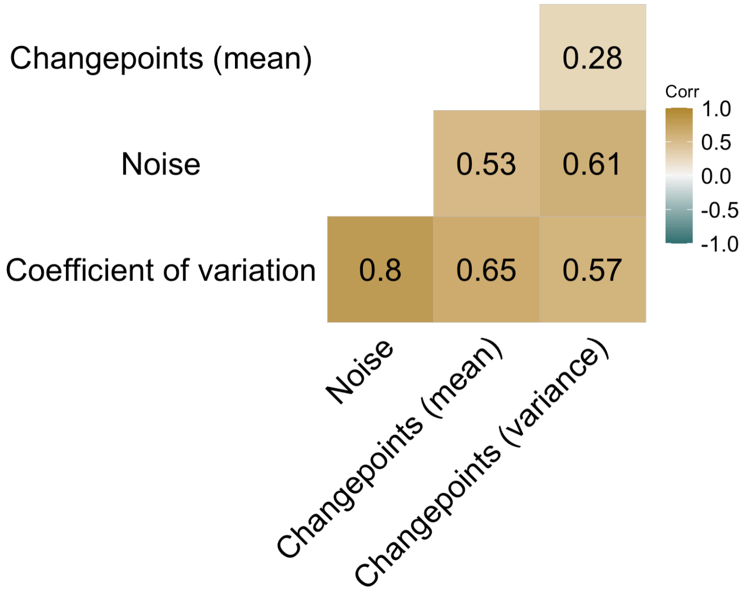

Figure S1. Spearman's rank correlations between unpredictability indices.

Additional information on the calculation of the noise index

During the revision process, we discovered an error in our computation of the (untransformed) noise statistic. The noise statistic summarizes the power spectral density of a time series at different frequencies. This frequency domain allows us to understand dynamic changes in the time series: We can quantify whether changes occur slowly (positive values) vs rapidly (negative values) and suddenly (small absolute magnitude) vs predictably (large absolute magnitude). When a time series is completely random (i.e. white noise), the power spectral density is equal across all possible frequencies, signifying a lack of any discernible patterns. Such a ‘perfectly’ random time series results in a noise value of zero. However, if there is no variability in a given time series (e.g., in a time series where all values are the same), power is zero at non-zero frequencies, also resulting in a noise value of zero. Our data contains such stable time series. Based on our initial noise computation, we would characterize these perfectly stable time series as completely unpredictable. This issue did not occur in previous work using this statistic, as there were never any time series without any variability.

To correct this mistake and for all noise calculations using our environmental statistics framework going forward, we now first identify all perfectly stable time series. Then we examine the histogram of (untransformed) noise values of the remaining data (Figure S2). We assign the maximum absolute noise value plus one to all perfectly stable time series (rounded to the nearest whole number). This ensures that perfectly stable time series receive the highest possible, positive (untransformed) noise value, indicating stability. As noise is an unbounded statistic (ranging from -infinity to +infinity), we could technically also assign +infinity (or a very large number) to these perfectly stable time series. However, this would lead to extremely distorted distributions with a huge gap between data points of participants with and without variability. This distortion would lead to artifacts in subsequent analyses.

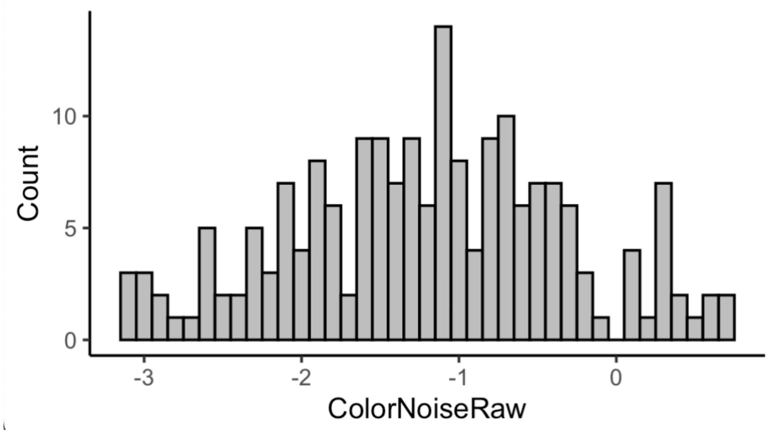

Figure S2. Histogram of noise (raw value).
